# EpiBOX: An Automated Platform for Long-Term Biosignal Collection

**DOI:** 10.3389/fninf.2022.837278

**Published:** 2022-05-23

**Authors:** Ana Sofia Carmo, Mariana Abreu, Ana Luísa Nobre Fred, Hugo Plácido da Silva

**Affiliations:** ^1^Department of Bioengineering, Instituto Superior Tecnico (IST), Universidade de Lisboa, Lisbon, Portugal; ^2^Instituto de Telecomunicações (IT), Lisbon, Portugal

**Keywords:** biosignals, data collection, data visualization, mobile technologies, health research, remote health

## Abstract

Biosignals represent a first-line source of information to understand the behavior and state of human biological systems, often used in machine learning problems. However, the development of healthcare-related algorithms that are both personalized and robust requires the collection of large volumes of data to capture representative instances of all possible states. While the rise of flexible biosignal acquisition solutions has enabled the expedition of data collection, they often require complicated frameworks or do not provide the customization required in some research contexts. As such, EpiBOX was developed as an open-source, standalone, and automated platform that enables the long-term acquisition of biosignals, passable to be operated by individuals with low technological proficiency. In particular, in this paper, we present an in-depth explanation of the framework, methods for the evaluation of its performance, and the corresponding findings regarding the perspective of the end-user. The impact of the network connection on data transfer latency was studied, demonstrating innocuous latency values for reasonable signal strengths and manageable latency values even when the connection was unstable. Moreover, performance profiling of the EpiBOX user interface (mobile application) indicates a suitable performance in all aspects, providing an encouraging outlook on adherence to the system. Finally, the experience of our research group is described as a use case, indicating a promising outlook regarding the use of the EpiBOX framework within similar contexts. As a byproduct of these features, our hope is that by empowering physicians, technicians, and monitored subjects to supervise the biosignal collection process, we enable researchers to scale biosignal collection.

## 1. Introduction

Biosignals, as direct and indirect measures of human physiology, represent a first-line source of information to understand the behavior and state of human biological systems. As such, they are extensively used in the field of medicine, as well as in the field of engineering, with applications ranging from healthcare to sports and quality of life, among others. Moreover, advancements in Machine Learning (ML) have precipitated their use as data sources to model the corresponding physiological systems (Alves et al., 2013; Semmlow, 2018), as well as the automation of analyses that are usually performed by physicians. While this automation does not replace human judgment, it can act as a convenient assistance to physicians, as well as an informative asset to patients outside the hospital environment.

Real-world scenarios in which ML is applied require the algorithms to detect patterns, learn from them, and execute tasks autonomously. Due to the innate complexity of human physiology, large volumes of collected data are needed to capture representative instances of all possible states. Several ML problems provide examples of the challenges faced in this field of research. The case of Epilepsy and seizure occurrence illustrates the issue of data representation: since seizure events are sporadic and difficult to predict, the large majority of collected biosignals pertains to non-seizure events resulting in unbalanced datasets. Moreover, common to all ML problems are the noise and artifacts introduced in the collected data by everyday activities. Being susceptible to a myriad of different activities, biosignals are exposed to a large variety of artifacts, once again requiring the acquisition of large volumes of representative data. Only then we can create healthcare-related algorithms that are both personalized and robust.

However, when relying solely on medical-grade equipment, data collection is constrained by the availability of equipment and resources for inpatient monitoring sessions. Besides removing the subjects from their ecological environment, this requirement severely restricts the duration of monitoring sessions which, consequently, limits the volume of data collected.

Discreet ambulatory equipment that can be seamlessly integrated into the subjects' day-to-day life provides a complementary solution to monitoring. Advances in the miniaturization of electronics and more powerful batteries allowed for the emergence of these flexible monitoring solutions (Yetisen et al., 2018), giving rise to ambulatory monitoring equipment that is both reliable and versatile. Holter monitors and more discreet frameworks such as Empatica's E4 wristband or Equivital's LifeMonitor aim exactly at that purpose. These devices can be worn outside the hospital environment, providing long-term acquisition of physiological data. Nonetheless, while some provide real-time visualization of the collected data, they are strictly designed for very specific applications, providing almost no customization in terms of the form-factor of the device or the biosignals being collected, which can be a significant constraint for research purposes. On the other hand, biosignal acquisition systems such as BIOPAC^1^ and BITalino (da Silva et al., 2014) address this issue by providing kits that can be easily adapted for all research needs. However, they often come with less intuitive frameworks that constitute a significant overhead for individuals with low technological proficiency to supervise the data collection process.

Therefore, we recognized the demand for an alternative framework for biosignal collection that automates most complex configurations and processes while still allowing for the same control and flexibility. The main objective is to scale the collection of biosignals by discarding the need for formal acquisition sessions in which supervision by researchers is required. By providing a standalone and automated platform that can be operated by individuals with low technological proficiency, acquisition can be performed on-site by physicians, technicians, or caregivers without it representing a burden (e.g., during already ongoing inpatient monitoring sessions or at the patients' homes). Therefore, the framework described in this paper aims at promoting cooperation between academia and the medical practice domain, enabling the scaling of biosignal collection for research purposes.

With this goal in mind, Section 2 starts off as an overview of what is currently available for researchers to expedite and scale biosignal collection. From this review, Section 3 establishes the requirements for the proposal of a new framework-EpiBOX. Within Section 4, the architecture and collection pipeline of the proposed system is described, also including some high-level notions of the implementation. Additionally, this section discusses some considerations regarding usability and system performance; and the results are assessed in Section 5. Finally, Section 6 provides some final thoughts and future considerations regarding the proposed system.

## 2. Related Work

Research within the physiological domain often relies on retrospective data acquired in hospital/clinical settings. However, it is a burdensome process, requiring the on-site extraction of large sets of data to external hard drives and generally implying the conversion of manual annotations to machine-readable formats. Moreover, regardless of recent attempts at standardization of data formats, structure and organization may be specific to each equipment or hospital software, hampering data handling (Halford et al., 2021).

With the rise of more flexible monitoring solutions, as described in Section 1, research has been quickly evolving from retrospective data recorded in a hospital/clinical setting, to data-intensive paradigms based on near-continuous and pervasive acquisitions (Yetisen et al., 2018). Within this scope, the proposed framework can be seen as an interface between the user and the biosignal acquisition system. In literature, three different methods can be identified that fit the same purpose: 1) computer software; 2) web-based platforms; and 3) fully-mobile solutions.

Computer software and web-based platforms may be seen as versatile and powerful solutions, accommodating computationally-heavy applications. Common programming frameworks often provide visualization interfaces that allow for the configuration and acquisition of biosignals. Khan et al. (2018) and Lin et al. (2021) proposed acquisition systems based on the MATLAB^®^ framework for the collection and online visualization of biosignals (although no code was publicly shared). Similarly, Polo et al. (2018) developed a graphical user interface based on LabVIEW^®^ for control and real-time visualization of the signals, although it was not clarified whether data was stored for later use. Alternatively, other computer software and web-based platforms have been proposed which are independent of any external programming frameworks. Alves et al. (2013) presented a web-based software framework for rapid prototyping of end-user applications called SignalBIT, aiming at a tradeoff between usability and performance. Likewise, OpenSignals^2^ also provides collection and real-time visualization of biosignals through a standalone software with some data analytics add-ons. Nevertheless, these platforms pose upfront a potential issue in long-term monitoring: having a computer/laptop running continuously may not be desirable in a personal setting or even feasible in restricted environments such as a hospital room due to space requirements.

As of now, as seen by the rise of smartphone and mobile application adherence, fully-mobile solutions are perhaps the most popular and attractive alternative for interaction with the user (Mehra et al., 2021), which has led to the development of some biosignal acquisition solutions such as MobileBIT (Cânovas et al., 2013) and OpenSignals mobile. In particular, Roy (2017) has found that users have significant switching intentions from computers/laptops to mobile applications, as a result of increased perceived usefulness and ease of use of this modality. Nevertheless, a limiting factor comes into play when appointing fully-mobile solutions as biosignal acquisition frameworks: the increase in battery consumption. By aggregating the operations of communication, data processing, storage, and visualization within a single smartphone, the burden on the device's battery is undeniable and can be seen as a bottleneck, particularly for long-term acquisitions. Moreover, even though the advances in chip technology have enabled on-device machine learning and artificial intelligence^3^, researchers are often more comfortable with employing their data processing and analysis algorithms in a language such as Python, which has several open-source libraries for biosignal processing and remains the most popular programming language^4^.

To our knowledge, there is currently no open-source solution that combines the computational power and flexibility of computer-based softwares with a mobile-based user interface, while still accommodating the possibility of integration of new acquisition devices and features. Therefore, with this work, we expect to support researchers with a versatile tool to record and visualize biosignals in long-term/ecological contexts, restricting the necessary technical knowledge to the minimum of everyday-/standard-use of a smartphone, thus expanding its use to physicians, technicians, and other users.

## 3. Proposed Approach

The primary need within our research group was to improve the process of long-term biosignal acquisition to enable physicians and other medical personnel to perform and supervise the whole process. Hence, in collaboration with our goal users, namely the Neurology department of Hospital de Santa Maria and Hospital Egas Moniz, Lisbon, Portugal, four core features were identified as mandatory to achieve the desired solution:

*(a) Enable users of all knowledge backgrounds to confidently perform data collection*
  Automated and minimally-assisted acquisition, coupled with an intuitive and user-friendly interface.
*(b) Real-time visualization*
  To verify that the acquisition setup is working as expected, the data being collected should be displayed in real-time.
*(c) Unobtrusive and portable design*
  From the perspective of end-users (both at home or inpatient clinical settings), the system must be easily set up in different locations and the hardware should be unobtrusive.
*(d) Compatibility, extensibility, and flexibility*
  From the perspective of an open-source tool for researchers, the framework should support a hassle-free extension of its functionalities and full reproducibility.

The first three requirements concern mostly the perspective of the end-user, aiming at an experience that empowers users of all knowledge backgrounds to perform the data collection. As such, requirement *(a) Usability* is fulfilled with PyEpiBOX—a Python software—which hides the intricacies of data communication, acquisition configuration, and storage within a software that automates every step.

Coupled with the software is the EpiBOX App—an Android mobile application, which provides an intuitive, user-friendly interface for the minimum-necessary interaction with the system. The EpiBOX App in turn also provides a near-to real-time visualization of all channels being acquired, thus fulfilling requirement *(b) Real-time visualization*.

Regarding requirement *(c) Portability*, the hardware involved in the framework is EpiBOX Core—composed of a Raspberry Pi as the autonomous recording unit, both operating the Python software and managing the data communication and storage—which acts as a practical and discrete alternative to a standard computer.

The major focus toward fulfilling requirement *(d) Compatibility* is on PyEpiBOX, developed with an *extensible* design, anticipating new features, different biosignal acquisition systems, and flexible storage locations. Moreover, all Bash/Python/Dart code is open-source, enabling reproducibility of the whole framework.

## 4. Methods

This section provides a high-level characterization of the proposed framework, including its architecture and data collection pipeline. An elementary description of the implementation of the framework is also given. Finally, the methodology used to assess the performance of the framework is described, which follows two different perspectives: user experience and data collection.

### 4.1. High-Level Architecture and Data Collection Pipeline

**Figure 1:**
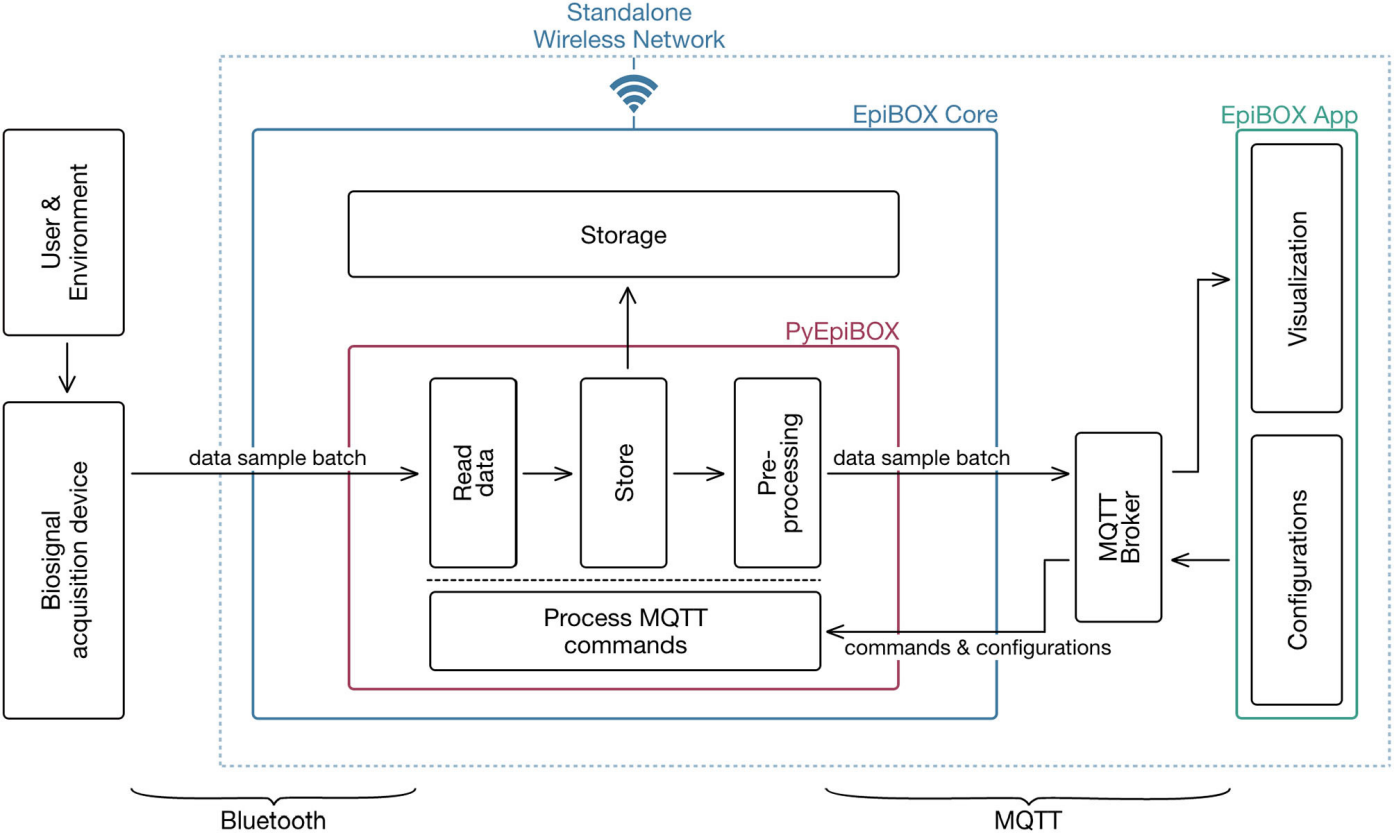
Architecture of the proposed biosignal acquisition framework. The three core components of EpiBOX are highlighted in blue (EpiBOX Core), bordeaux (PyEpiBOX) and green (EpiBOX App). Within the PyEpiBOX module, the main features/functions are illustrated. The standalone WiFi network is deployed by EpiBOX Core and holds the communications performed through MQTT (communications on the right). Contrarily, the communication between the biosignal acquisition system and EpiBOX Core is held through Bluetooth (communication on the left). Although the preprocessing step is not described in the paper, it is provided in the Supplementary Material.

EpiBOX is composed of three elements as illustrated in Figure 1: EpiBOX Core, PyEpiBOX and the EpiBOX App. While the biosignal acquisition system is not part of the framework, it is required for the collection of biosignals^5^. The pipeline of data collection is also illustrated, however in a simplified manner. The remainder of this section describes the several considerations taken within the development of the proposed framework, following the pipeline of biosignal collection: from initiation and data exchange to storage and visualization.

#### 4.1.1. Initiating Data Collection

EpiBOX fully automates the data collection process: PyEpiBOX is continuously attempting to connect to the default biosignal acquisition devices. Therefore, an acquisition session with the default configurations is initiated once the user turns on the default acquisition devices.

Nonetheless, set up of the default devices and acquisition configurations can be performed in the EpiBOX App, prior to initiating data collection.

#### 4.1.2. Data Exchange for Signal Recording: Acquisition System-PyEpiBOX

Once the connection between the acquisition system and PyEpiBOX is established, data is exchanged through Bluetooth wireless data transmission. This communication protocol is a common practice for low-power, low data-rate devices (Abedi et al., 2019), seen in IoT-based devices such as BITalino, Arduino and MySignals^6^. Through a Python API (specific to the acquisition system in use), PyEpiBOX reads the data samples that were received via Bluetooth.

#### 4.1.3. Data Storage

**Figure 2:**
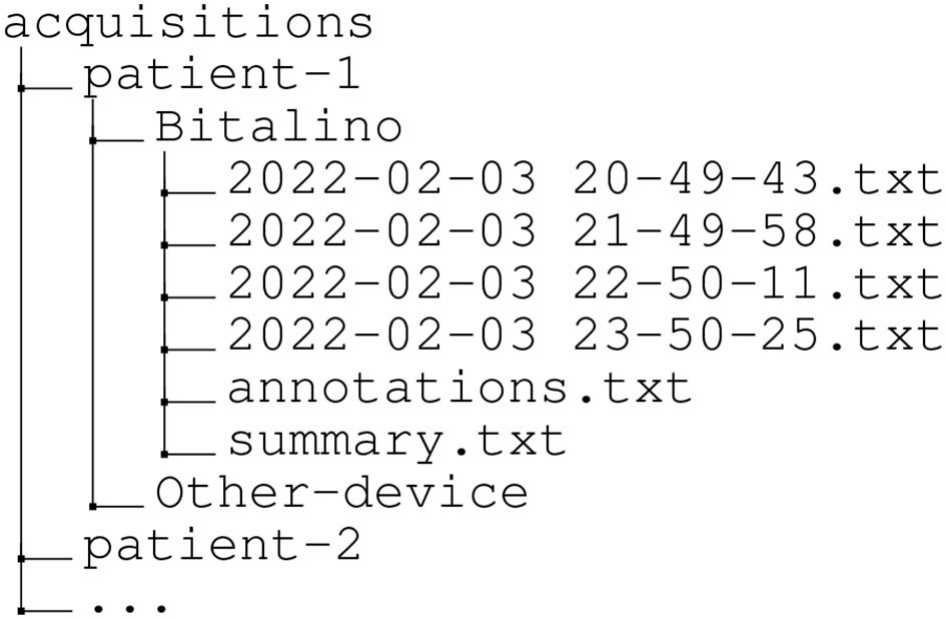
Directory tree illustrating organization of the collected data, highlighting a patient-based structure. Besides the acquisition files, two more files are provided for each patient ID, including the annotations performed by the user and a summary of the duration of each acquisition session.

Once a batch of data samples is read by PyEpiBOX, it is written to the active session's *.txt* file. This file format is common practice in open-source systems such as BITalino and Arduino, as it provides human-readable notation^7^. Each session's file is comprised of a header (in dictionary format, with information regarding the acquisition configurations and column mapping) and several tab-separated values (where each row corresponds to a sample). Files are identified with the indication of the session's date and starting time (e.g., YYYY-MM-DD HH-MM-SS.txt) and are structured in a patient-based organization as illustrated in Figure 2.

Moreover, since EpiBOX is designed for long-term acquisitions, to prevent the creation of files with unmanageable volumes, the acquisitions are segmented into 1-h-long sessions, enabling better manageability of the data in post-processing.

Regarding storage location, according to the type of use case scenario, researchers might wish to store the data in a remote server (cloud storage) to have remote access to the collected data. Alternatively, for example, when an internet connection is not available (or undesirable), the solution lies in resorting to an external hard drive connected to EpiBOX Core. As a use case, and for illustrative purposes only, this work describes the implementation of data storage in (local) external hard drives only, enabling the acquisition of data in clinical environments where access to the internet is restricted, while also minimizing the exchange of sensitive data. Nevertheless, if remote access to the collected data is required, by using secure protocols, such as the SSH File Transfer Protocol (SFTP), data can be safely transferred to secure storage locations such as Network-Attached Storage Devices (NASs). These NAS systems allow for storage and retrieval of data from a centralized location by authorized users only, adding an additional security layer.

#### 4.1.4. Data Exchange for Visualization: PyEpiBOX-EpiBOX App

Following the storage procedure, the batch of samples is sent to the EpiBOX App for visualization purposes. Nevertheless, in case the user is not using the EpiBOX App, the acquisition and storage procedures continue as normal.

Given the limitations of the Bluetooth protocol regarding streaming capacity^8^, instead of further overloading the Bluetooth network with another connection, the communication between PyEpiBOX and EpiBOX App is alternatively established via a standalone WiFi network.

**Figure 3:**
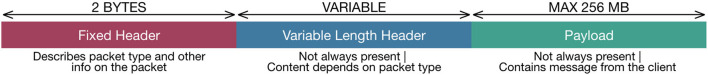
Illustration of the structure of an MQTT message packet.

Due to the structure and lightweight nature of the data exchanged between both devices, communication is performed through the MQTT communication protocol. In this protocol, each communication event corresponds to the exchange of a packet that contains the payload (actual message) and packet overhead (Figure 3), as in Bluetooth. However, this type of communication is not limited to two devices, adopting a client-server architecture. Compared to other communication protocols, such as the widely used HTTP, MQTT has an appealing feature, as it ensures high delivery guarantees and provides customization of delivery service quality level. As such, it is optimized for high-latency or unreliable networks. Moreover, this protocol has its own security-assuring methodologies, including a procedure of “identity, authentication, and authorization”^9^, increasing the security of sensitive data exchange.

Once the batch of samples is received by the EpiBOX App, it is displayed in near-real-time—and the collection/visualization pipeline is complete. To avoid the storage of sensitive medical data on a smartphone, once displayed, data samples are immediately discarded.

### 4.2. Implementation

The proposed framework entails a threefold development, corresponding to each core element of EpiBOX, i.e., EpiBOX Core, PyEpiBOX, and EpiBOX App. The remainder of this section provides an elementary description of their implementation. Nevertheless, further considerations are supplied in the Supplementary Material.

#### 4.2.1. EpiBOX Core

To establish the MQTT communication channel, EpiBOX Core is responsible for providing a network (considering that WiFi might not necessarily be available in the experiment environment), as well as an MQTT server. To do so, EpiBOX Core supports two functionalities: (1) it is set up as a standalone WiFi access point, thus supporting wireless services and allowing access by other wireless devices (Liu et al., 2020); and (2) it is configured as an MQTT broker, providing a password-protected connection to potential clients within the network.

EpiBOX Core is also responsible for automatically launching PyEpiBOX, which is ensured through systemd—a Linux system and service manager. All developments regarding the setup of the Linux requirements and services are established using Bash.

#### 4.2.2. PyEpiBOX

The acquisition software is assured by PyEpiBOX^10^, which is entirely developed using the Python language. Integration with the biosignal acquisition system is provided through a Python API. Having compatibility and extensibility as design requirements, to adapt the platform to other open-access biosignal acquisition devices, only minor adaptations are required in PyEpiBOX (i.e., substituting API commands). Alternatively, if the device does not provide a Python API, there are integration solutions that provide interoperability between programming languages.

#### 4.2.3. EpiBOX App

The EpiBOX App was developed using the Dart language under a high-level programming framework called Flutter and is designed to maximize intuitive use and flexibility. The main screen has four interchangeable tabs, that enable the user to freely explore the functionalities (e.g., checking configurations during an ongoing acquisition). Nevertheless, EpiBOX App also prevents the user from skipping steps or mistakenly performing actions out of order. Supplementary Figure S1, which can be found in the Supplementary Material, illustrates a standard interaction with the mobile application.

Through the EpiBOX App, full customization of the data collection is provided: from the default biosignal acquisition devices (Supplementary Figure S1B) and the respective analog channels/sensor labels to the desired sampling frequency and storage location (Supplementary Figure S1C). In turn, the visualization screen (Supplementary Figure S1D) has its own set of tabs, according to the number of biosignal acquisition devices, and renders a plot for each channel—which is updated as new data points are received. Moreover, it provides controllers that allow the user to manually pause/resume/terminate the acquisition freely, as well as record short annotations along with the acquisition files (e.g., labeling events within the acquisition).

#### 4.2.4. Open-Source Development

All the source code for the developments described in Section 4.2 is available under the MIT license. The source code for the EpiBOX App and PyEpiBOX is hosted on GitHub under PIA-Group/epibox_app and PIA-Group/epibox, respectively. The full bash script to reproduce EpiBOX Core on a Raspberry Pi can be found at https://drive.google.com/drive/folders/1i9GuQ9RFtaadw_9xBPMq8NWy_U5VQR81?usp=sharing. For convenience, the image of the Raspberry Pi with all configurations in place can also be found at the same address^11^.

### 4.3. Experimental Methodology for Technical Evaluation

In a framework such as EpiBOX, user experience is highly determined by response time^12^, CPU/energy consumption, and rendering performance. While none of these factors have any impact on the data stored for research purposes, they do have the potential to provide a more or less pleasant experience for the user that is supervising the acquisition using the EpiBOX App. Considering this, two factors were identified as determinants for user experience: *(i) Data transfer latency* and *(ii) Performance of the EpiBOX App*.

#### 4.3.1. Data Transfer Latency

Unexpected delays within the MQTT data transfer can affect either the response time (e.g., when the response is dependent on an MQTT message reply) or the visualization (e.g., if there is a perceivable delay mid-acquisition, it will result in non-smooth rendering), ensuing an unpleasant user experience.

Considering that this factor is dependent on the size of the MQTT messages, it can be influenced by the number of channels/devices in acquisition. To infer the effect of these variables, a stress test was performed under three use cases, contemplating the use of (1) one acquisition device in the minimum channel configuration-1 channel; (2) one acquisition device in maximum channel configuration-6 channels; and (3) two acquisition devices in maximum channel configuration-12 channels.

Besides the size of the MQTT messages, other variables can also affect factor *(i) Data transfer latency*, namely the Received Signal Strength (RSS). The RSS provides a measurement of received WiFi signal strength, accounting for factors such as the distance between the WiFi router and the MQTT client, as well as the existence of physical obstacles between them. Given that RSS can effectively influence the throughput of data exchange through MQTT (Chou et al., 2009), while it can not impact data collection it can impact visualization. Therefore, another stress test was performed, accounting for RSS as a variable.

Different experimental configurations were explored by adjusting the router-client distance and room layout to achieve three approximate RSS test values: 100, 80, and 50%^13^ (a more in-depth description of the experimental setup is provided in the Supplementary Material).

#### 4.3.2. Performance of the EpiBOX App

Expensive Dart code and unnecessary widget rebuilds can overload the GPU and rendering performance, resulting in *jank* (i.e., skipped frames). According to the Flutter guidelines, to ensure the standard rendering rate of 60 frames per second (fps) the time to render a frame should not exceed 1/60th of a second (i.e., approximately 16 ms)^14^. Moreover, unexpected delays caused by lengthy building/rendering processes can cause unease/anxiety for the user (e.g., large mobile application startup time). Additionally, unused resources/libraries and uncompressed images can unnecessarily increase the size of the mobile application.

The performance of the EpiBOX App was assessed through an integration test framework, which allows for a standard and reproducible benchmarking, providing metrics for jank (i.e., missed frames), download size, and startup time. As such this framework was used as a tool for performance profiling, namely addressing factors *(ii) Dart code performance* and *(iii) App size*.

The script for the integration test (available at the GitHub repository) performs a standard operation of the EpiBOX App. It assumes that the connection to the acquisition devices was successful on the first trial (if not, it will continue but it will not display the real-time visualization), contemplating an acquisition period of 30 min. To evaluate the performance of the EpiBOX App in diverse conditions, two smartphone devices with different specifications (Alcatel 1SE and Huawei Mate 10 Pro) were tested under the same use case, corresponding to the configuration of one acquisition device, with six channels, and the real-time display of the acquired data with the screen on.

Another very relevant matter in mobile performance testing is battery efficiency. In a mobile application, the biggest sources of power usage are CPU intensive calls, complex video rendering, and network calls (AppDynamics, 2014). However, in iOS and Android, this is a particularly challenging metric to obtain considering that the speed of CPU and GPU units are adjusted autonomously according to load, to obtain a smooth performance while consuming the minimum possible battery^15^. As such, instead of measuring battery consumption as an approximation of CPU time, the built-in feature of energy consumption on the smartphone was used.

## 5. Results and Discussion

This section discusses the results obtained during the experiment described in Section 4.3 and provides a use-case scenario for EpiBOX.

### 5.1. Technical Performance and Limitations

#### 5.1.1. Data Transfer Latency

As described in Section 4.3.1, two experiments were performed: one concerning the number of acquisition channels and another concerning the RSS. For each experiment, an acquisition of 5 min was performed, resulting in approximately 3,000 MQTT messages exchanged and recorded.

**Figure 4:**
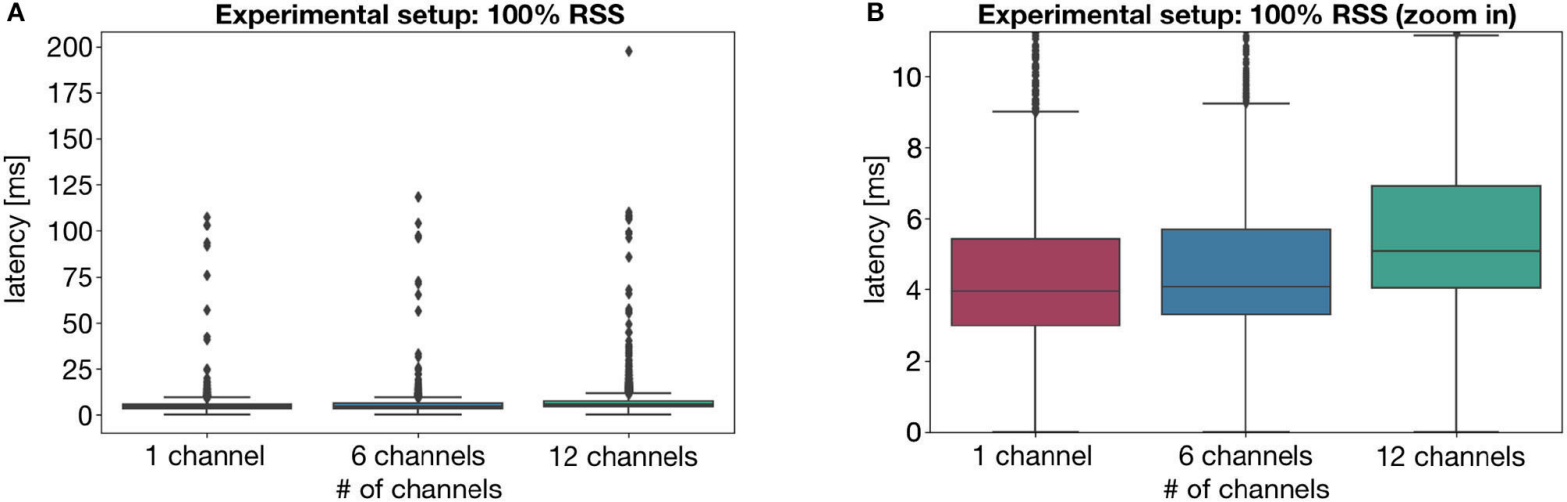
Visual representation of the latency values for the experiment setup with 100% RSS, showing **(A)** all latency values and **(B)** a portion of the plot, disregarding the outliers. Values obtained for three acquisition configurations: 1 acquisition device-1 channel; 1 acquisition device-6 channels; and 2 acquisition device-6 channels each.

**Figure 5:**
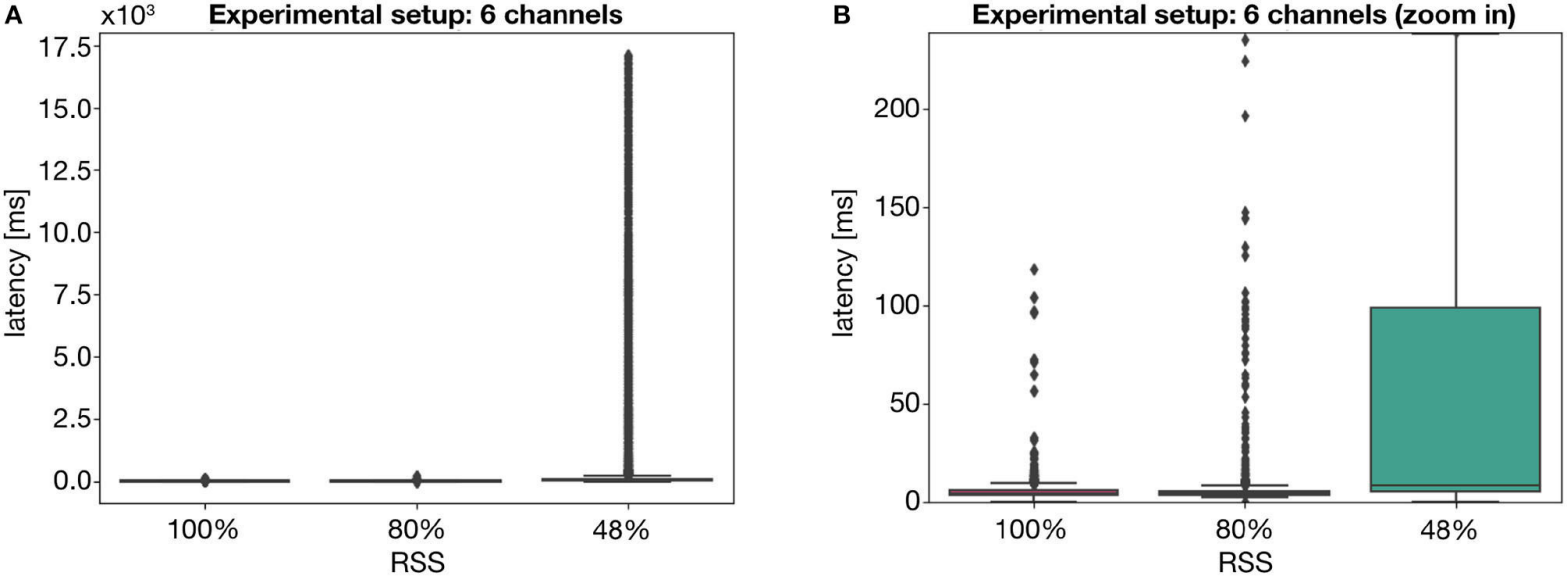
Visual representation of the latency values for the experiment setup with 1 acquisition device, with 6 channels, showing **(A)** all latency values and **(B)** a portion of the plot, disregarding the outliers. Values obtained for three acquisition setups: 100% RSS, 80% RSS and 50% RSS.

**Figure 6:**
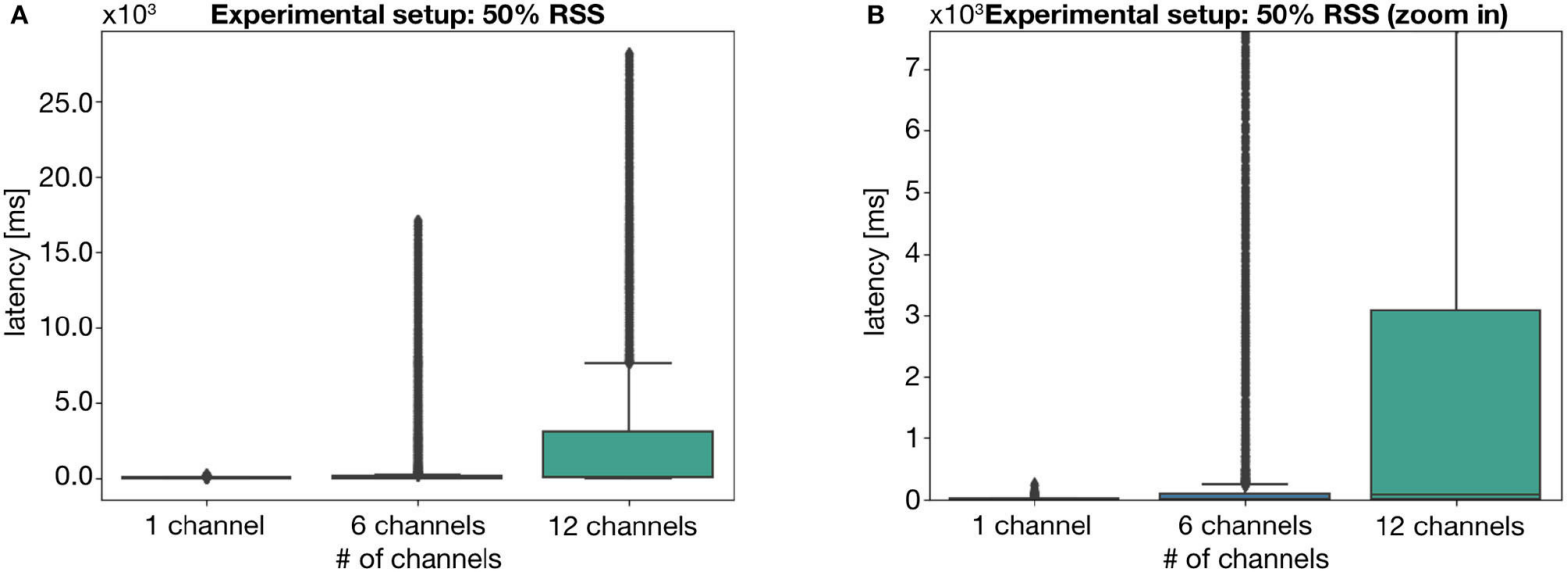
Visual representation of the latency values for the experiment setup with approximately 50% RSS, showing **(A)** all latency values and **(B)** a portion of the plot, disregarding the outliers. Values obtained for three acquisition configurations: 1 acquisition device-1 channel; 1 acquisition device-6 channels; and 2 acquisition device-6 channels each.

Table 1 shows the latency results obtained for the experiments, with the channels under acquisition and approximate values for the respective RSS. Moreover, Figures 4–**6** provide a visual interpretation of some of the results, via a boxplot representation.

**Table 1:** Median values, in milliseconds, for the latencies measured in the experiments described in Section 4.3.1.

		**RSS**		
		**100%**	**80%**	**50%**
# Channels	1	4 ms	4 ms	6 ms
	6	4 ms	4 ms	8 ms
	12	5 ms	5 ms	74 ms

*RSS, Received Signal Strength*.

As shown in Figure 4, with maximum signal strength, there is virtually no impact on the latency by acquiring an additional 5 channels. However, this impact becomes slightly more evident when a total of 12 channels is acquired, resulting in a higher median value, as well as a larger amplitude of latency values. Nevertheless, the maximum latency is still significantly low. Interestingly, these outcomes are also supported by the results achieved for an RSS of 80%. However, the same can not be suggested when the RSS falls near to the minimum required for stable communication. As supported by Figure 5, such a low RSS results in significantly larger latencies, even for a 6 channel acquisition—achieving, at one point, more than 10 s of latency.

This becomes even more evident when combining the impact of both weak signal strength and a larger number of acquired channels, as shown in Figure 6. The joint effect of a low RSS and a larger MQTT packet results in an incomparable median latency of 74 ms, as well as a maximum value of more than 25 s. As such, we can assert the individual and combined impact of these two external factors on the latency of MQTT messages which, at times, may hinder the user experience.

Alongside these statements, it is relevant to express that the authors find these results encouraging. In fact, particularly for RSS values of 100% and 80%, the latency values remain innocuous regarding visualization purposes. Even for a signal strength below what is required for stable communication (which should not be the case for any real-life applications) the message transfer time is still manageable for the majority of the communication events.

#### 5.1.2. Performance of the EpiBOX App

Table 2 describes the performance results achieved for the experiment described in Section 4.3.2, as well as the specifications of the smartphones tested. These results serve as an illustrative example of what the user can expect if a smartphone with similar specifications is used.

**Table 2:** Performance results for the experiment performed using the integration test framework, for a 30 min acquisition with one acquisition device.

**Device**	**Specifications**			**Performance**			
	**RAM**	**Processor**	**Android**	**Startup**	**App**	**Battery**	**Build and**
			**Version**	**time**	**size**	**usage**	**Rendering times**
**(A)**	3.0 GB	Spreadtrum	10	926 ms	20.92 MB	–	*avg:* (5.9 + 14.3) ms
							*90th:* (6.7 + 15.1) ms
**(B)**	6.0 GB	Kirin 970	10	235 ms	21.48 MB	81.84 mAh	*avg:* (3.8 + 9.1) ms
							*90th:* (4.2 + 10.0) ms

*(A) Alcatel 1SE; (B) Huawei Mate 10 Pro. Startup Time: time until the first frame of EpiBOX App is rendered. Build and Rendering Times: average and 90th percentile times for the whole experimental acquisition, given as (build time + rendering time)*.

We can observe the slight susceptibility of the analyzed metrics to the smartphone's specifications - in general, smartphone (B) presented more satisfactory results than its counterpart. Nevertheless, both devices successfully launched the first screen of the mobile application within a reasonably short time; as well as displaying very reasonable build and rendering times, even for the 90th percentile, always remaining below the recommended reference time values (thus guaranteeing the 60 fps).

Regarding battery usage, although device (A) did not contain a built-in tool for measuring this parameter, device (B) provided the indicative value of 81.84 mAh.

In summary, these results illustrate that satisfactory performance of the EpiBOX App can be achieved with smartphones from two very different price points (with significantly different RAM and processors), accommodating a large spectrum of users.

### 5.2. Real-World Evaluation: A Use Case

#### 5.2.1. Use Case Description

The experience of the authors in Epilepsy research provides a standard scenario for the use of EpiBOX within the research scope described in this work.

To explore some of the autonomic effects of seizures, the authors rely on a BITalino-based chestband for the acquisition of single-lead Electrocardiography (ECG), 3-axis acceleration, and the displacement variations induced by inhaling/exhaling. In a standard acquisition session, the experimental setup consists of the EpiBOX framework and one chestband (comprised of two dry-electrodes that are in direct contact with the patient's skin, an ECG sensor, a Piezoelectric Respiration (PZT) sensor, and an Accelerometer (ACC) sensor).

Under the collaborations with the two Portuguese hospitals, data collection is performed during inpatient sessions, while the patients are under video-Electroencephalography (video-EEG) monitoring. The previous data collection setup required the use of a standard portable computer in the patient's monitoring room which was a significant concern due to space constraints. Additionally, the configuration and initiation of the acquisition session were performed by running a Python script directly on a terminal window. This constituted a significant overhead for individuals with low technological proficiency to handle data collection. As such, the previous setup required daily visits to the hospitals by one of the authors to replace the batteries of the acquisition devices and restart data collection.

Since EpiBOX was introduced to both Portuguese hospitals, data collection is fully operated by the technicians responsible for inpatient monitoring. This was made possible due to the full automation of the acquisition process: the technicians are only required to replace the battery on the device once a day, releasing them from any additional intervention or interaction with EpiBOX. This constituted a significant optimization of the data collection process since the monitoring sessions require no active supervision on the authors' behalf. Moreover, EpiBOX Core is much smaller and more discreet than a standard portable computer, further contributing to a pleasant user experience.

#### 5.2.2. Real-World Evaluation

The duration of the acquisition sessions varies according to each individual case, ranging from 1 to 5 days (*M* = 2.7 days, *SD* = 1.2 days). Data volume, on the other hand, varies to a greater extent ranging from 16 MB to 6 GB (*M* = 2.6 GB, *SD* = 2.7 GB).

In an attempt to characterize the volume of data that is not captured due to processing times within the EpiBOX pipeline, a controlled experiment was performed. This experiment consisted of an 85-h-long acquisition (3 days and 13 h) with one chestband, i.e., 5 channels acquired at a sampling rate of 1,000 Hz. Besides an initial setup of the default device and configurations on the EpiBOX App, the only human interventions that took place were 3 changes of battery (one every 24 h, approximately). Furthermore, this experiment provides a characterization of EpiBOX under a standard use scenario, establishing the behavior of the framework after the end of each session.

The 85-h-long acquisition (3 days and 13 h) resulted in a total of 159 acquisition files (compared to the 88 expected), indicating a larger number of interruptions than anticipated. Regarding file size, the complete acquisition summed to 5.45 GB, with a median value of 34.77 MB per file (*M* = 35.1 MB, *SD* = 29.78 MB).

Although the number of interruptions was larger than anticipated, the actual number of interruptions is not determined by the performance of EpiBOX but rather by the stability of the Bluetooth connection - once the connection between the acquisition system and EpiBOX Core is lost, the acquisition session is terminated. Nevertheless, one can comment on this result: if instead, we look at the average number of interruptions per hour, it is rather close to the expected (*M* = 1.8 interruptions/h, *SD* = 2.8 interruptions/h), presenting a median of exactly 1 interruption/h.

On the other hand, the duration of these interruptions, while still susceptible to the impact of Bluetooth stability, can be more indicative of the performance of the framework. In fact, the time between the interruption of the Bluetooth connection and the start of a new acquisition can lead to the loss of large volumes of data if the processes within PyEpiBOX are not optimized.

The results of the experiment indicate an average duration of the interruptions of 64.8 s (*SD* = 320.2 s) and a median of 14.2 s. While these results may seem large, the median duration is satisfactory, corresponding to an almost insignificant 0.4% data loss of a 1-h long session (if a single interruption occurs). As such, these results illustrate a promising performance outlook while still presenting space for improvement.

## 6. Conclusion

This paper provides a detailed description of EpiBOX, an open-source, standalone, and automated platform that enables the long-term acquisition of biosignals, passable to be operated by individuals with low technological proficiency. As it was initially designed for the clinical context, with restricted access to the internet, EpiBOX currently allows storage on external hard drives only. However, with the continued use and inherent evolution of the system, other developments will unfold, evidently addressing issues such as remote storage. Moreover, this paper provides an in-depth analysis of the performance of the system, with a focus on the perspective of the end-user, tackling the subjects of communication delays, mobile application performance, and data loss. The results achieved within the evaluation indicate a suitable performance in all aspects, providing an encouraging outlook on adherence to the system.

This framework builds upon previous work of our research group, emerging with the ultimate goal of accelerating the development of ML solutions in health. At the time of completion of this paper, the EpiBOX framework was successfully integrated into two Portuguese hospitals, providing two favorable use case examples. At this stage, our expectation is that EpiBOX promotes the collaboration between researchers and physicians, technicians and monitored subjects, allowing for the expansion of the collection of data, and thus bridging the gap between their theoretical approaches and the implementation of real-world solutions.

## Data Availability Statement

All the source code for the developments described in this work is available under the MIT license. The source code for the EpiBOX App and PyEpiBOX are hosted on GitHub under PIA-Group/epibox app and PIA-Group/epibox, respectively. The full bash script to reproduce EpiBOX Core on a Rapsberry Pi can be found at https://drive.google.com/drive/u/1/folders/1i9GuQ9RFtaadw_9xBPMq8NWy_U5VQR81. For convenience, the image of the Raspberry Pi with all configurations in place can also be found at the same address.

## Author Contributions

AC and MA developed the tool and ran the experiments. AC wrote the article. HS and AF reviewed the article. All authors contributed to the conceptualization of the solution and to the design of the experimental evaluation.

## Funding

This work has been partially funded by the Fundação para a Ciência e Tecnologia (FCT), Portugal, under the scholarship 2021.08297.BD and under the project PCIF/SSO/0163/2019 SafeFire, by the FCT/Ministério da Ciência, Tecnologia e Ensino Superior (MCTES) through national funds and when applicable co-funded by EU funds under the project UIDB/50008/2020, by the Instituto de Telecomunicações (TI) under grant BI/N°16-16/03/2021, and by Centro Hospitalar Universitário Lisboa Norte, EPE, under the project Pre_EpiSeizures.

## Conflict of Interest

The authors declare that the research was conducted in the absence of any commercial or financial relationships that could be construed as a potential conflict of interest.

## Publisher's Note

All claims expressed in this article are solely those of the authors and do not necessarily represent those of their affiliated organizations, or those of the publisher, the editors and the reviewers. Any product that may be evaluated in this article, or claim that may be made by its manufacturer, is not guaranteed or endorsed by the publisher.

## Footnotes

^1^BIOPAC: https://www.biopac.com/product-category/research/systems/mp36r-4-channel-systems/.

^2^OpenSignals: https://bitalino.com/downloads/software.

^3^Android Authority. “How on-device machine learning has changed the way we use our phones.” (Online, accessed November 2021).

^4^Statista, 2020. “Python Remains Most Popular Programming Language” (Online, accessed November 2021).

^5^As a use case, and for illustrative purposes only, this work describes the use of EpiBOX with a specific open-source platform - BITalino.

^6^For details please see https://www.cooking-hacks.com/documentation/tutorials/ehealth-biometric-sensor-platform-arduino-raspberry-pi-medical.html.

^7^Nevertheless, adapting storage to other data formats available in Python is straightforward and may be desired for some applications.

^8^Foley, 2007. “How Does Bluetooth Work?.” (Online, accessed July 2021).

^9^IBM. “MQTT Security.” (Online, accessed March 2022).

^10^Python package available on PyPI at https://pypi.org/project/epibox/.

^11^As a proof-of-concept, the framework was designed and tested with a Raspberry Pi 4 and BITalino-based devices. As such, when using this equipment, the provided source code enables off-the-shelf use.

^12^Response time corresponds to the time interval between an interaction with the user interface and the corresponding system response.

^13^The minimum RSS under test was 50%, as this is the minimum required to ensure a stable communication. "Wi-Fi Signal Strength: What Is a Good Signal And How Do You Measure It". EyeNetworks [Online, accessed October 2021].

^14^For details please see https://flutter.dev/docs/perf/rendering/ui-performance.

^15^Medium. "Performance testing of Flutter apps" for more information on this [Online, accessed October 2021].
