# Supplementary material for: EpiBOX: An Automated Platform for Long-Term Biosignal Collection
Source: Front Neuroinform. 2022 May 23;16:837278. doi: 10.3389/fninf.2022.837278 (PMC9168798; doi:10.3389/fninf.2022.837278)
Supplement: Supplementary file 1 [file Data_Sheet_1.PDF]

Supplementary Material

1 EPIBOX CORE: IMPLEMENTATION CONSIDERATIONS

The communication between EpiBOX Core and EpiBOX App is held through a Message Queue Telemetry Transport (MQTT) protocol, which comprises two types of entities within a network: the clients and the broker. Since WiFi might not necessarily be available (or desirable) in the experimental environment, this implies the creation of a network and a server. As expected, EpiBOX Core is responsible for providing both functionalities:

(i) *EpiBOX Core as a Wireless Access Point*
Acting as a standalone WiFi access point, wireless services are supported and access by other wireless devices is enabled (Liu et al., 2020).

(ii) *EpiBOX Core as an MQTT Broker*
Operating as an MQTT broker, a password-protected connection is provided to potential clients within the network.

1.1 Setting up EpiBOX Core as a Wireless Access Point

Setting up EpiBOX Core as an access point requires some configuration[1], which is aggregated in an executable Bash script for convenience. This setup process requires the `dnsmasq` package, as well as the `hostapd` package, consisting of the following steps:

(i) *Configuring a static IP for the `wlan0` interface*
Which guarantees that we always know the IP address of our EpiBOX Core, even when it is connected to different wired networks.

(ii) *Configuring the DNS, DHCP server*
Using the `dnsmasq` package, it allows the allocation of IP addresses to the connected devices, enabling them to be on a separate subnet from the wired network.

(iii) *Configuring the access point host software*
Using the `hostapd` package, it configures the access point (e.g. defines the network ID and password).

(iv) *Setting up traffic forwarding*
Provides Internet access to the connected devices when EpiBOX Core is connected to a network over Ethernet.

(v) *Configuring Network Address Translation between the Ethernet and WiFi interfaces*
Allows devices on both networks to communicate with each other.

1.2 Setting up EpiBOX Core as an MQTT Broker

There are several MQTT brokers available, including both free and commercial options, such as *Eclipse Mosquitto* which is one of the most popular[2].

[1] For details, please see `https://www.raspberrypi.org/documentation/computers/configuration.html#setting-up-a-routed-wireless-access-point` and `https://learn.sparkfun.com/tutorials/setting-up-a-raspberry-pi-3-as-an-access-point/enable-packet-forwarding`

[2] For details please see `https://mosquitto.org/`

Mosquitto is an open source MQTT broker that runs on Windows and Linux, and provides easy-to-implement client capabilities for both Python and Flutter. Moreover, it also also requires minimum effort for setting up the broker for the first time since it only implies the installation of the `mosquitto` package and configuration of the client authentication options.

1.3 Launching PyEpiBOX

The proposed framework is designed to work with a headless Raspberry Pi[3] in an automated fashion, which requires it to set up and launch all requirements on boot, as well as running the necessary services/scripts.

When EpiBOX Core is turned on, it automatically launches a service (`EpiBOXStartup`) that is responsible for executing a Bash script (`epibox_startup.sh`), as presented below:

```
[Unit]
Description=EpiBOXStartup
After=network-online.target

[Service]
Type=idle
ExecStart=/home/pi/bin/epibox_startup.sh
User=pi
WorkingDirectory=/home/pi
Restart=always
RestartSec=2

[Install]
WantedBy=multi-user.target
```

There are two main configurations of the service that allow for seamless use in the context of automated data collection:

(i) `After=network-online.target`
Guarantees that the network is set up before launching the service.

(ii) `Restart=always`
Causes the service to be re-launched every time it is terminated (i.e. after the end of an acquisition, thus enabling the start of a new acquisition process).

In turn, `epibox_startup.sh` runs PyEpiBOX, initiating the acquisition process and enabling the communication with EpiBOX App. Whenever an acquisition is concluded, PyEpiBOX is terminated and the service `EpiBOXStartup` is relaunched.

[3] Headless setup: when the Raspberry Pi is not connected to a monitor or keyboard.

2 PYEPIBOX: IMPLEMENTATION CONSIDERATIONS

When a batch of samples is received by PyEpiBOX, it is stored and prepared to be sent to the EpiBOX App for visualization purposes. Since this processing is not performed on the stored data, subsampling can be performed in order to reduce batch size, as well as the energy consumption while rendering the sample points on the EpiBOX App. Moreover, as the number of pixels available for drawing the time series on the mobile application varies according to the smartphone used, subsampling also allows to visualize longer intervals of the biosignals.

Therefore, if the data is acquired at a sampling frequency of 1000 Hz, it is subsampled through decimation. This technique differentiates from regular subsampling (i.e. removing samples from the signal) by avoiding aliasing. Aliasing occurs when the signal contains frequencies higher than the Nyquist frequency (half the sampling rate) and, therefore, the sampling can not retain all the characteristics of the signal, causing distortion[4].

The first step within decimation is an 8th-order Chebyshev type I filter (with phase shift prevention as described in `scipy`'s function `decimate`). This process limits the frequency content of the signal to below the Nyquist frequency, preventing aliasing during the subsequent subsampling. As a second (and final step), the filtered signal is downsampled by a factor of 10.

3 USER CONSIDERATIONS: EXPERIMENTAL SETUP

Determining the latency of the communication within a particular communication channel is done by computing the time between a message being sent from one client until it is received by the other client. However, since clock synchronization between two devices is difficult to guarantee, in this experimental setup latency was measured by a single device: since it acts both as a publisher and subscriber, it is possible to record a timestamp right before a message is sent and another timestamp right after it is received through the MQTT channel.

Evidently, this methodology assumes a symmetric transfer time from the publisher to the broker and from the latter to the subscribers, which, in regular circumstances, is not the case. As an illustrative example, in the standard use-case of when EpiBOX Core and PyEpiBOX are set up in the same device, there is no distance or physical obstacles between the client (PyEpiBOX) and the MQTT broker (EpiBOX Core) - therefore Received Signal Strength (RSS) would be 100%. As an alternative, if another Linux device is used as a client instead, this methodology allows us to study the potential impact of MQTT message size and network signal strength on communication latency, while performing an estimation by excess.

[4] Aliasing can also be explained in the frequency domain: when the signal is sampled at an inadequate frequency, there is overlap of the copies of the signal spectrum, adding them together, which causes corruption of the frequency content and distorts the signal. For details please see `https://www.mathworks.com/help/signal/ug/downsampling-aliasing.html`

4 SUPPLEMENTARY TABLES AND FIGURES

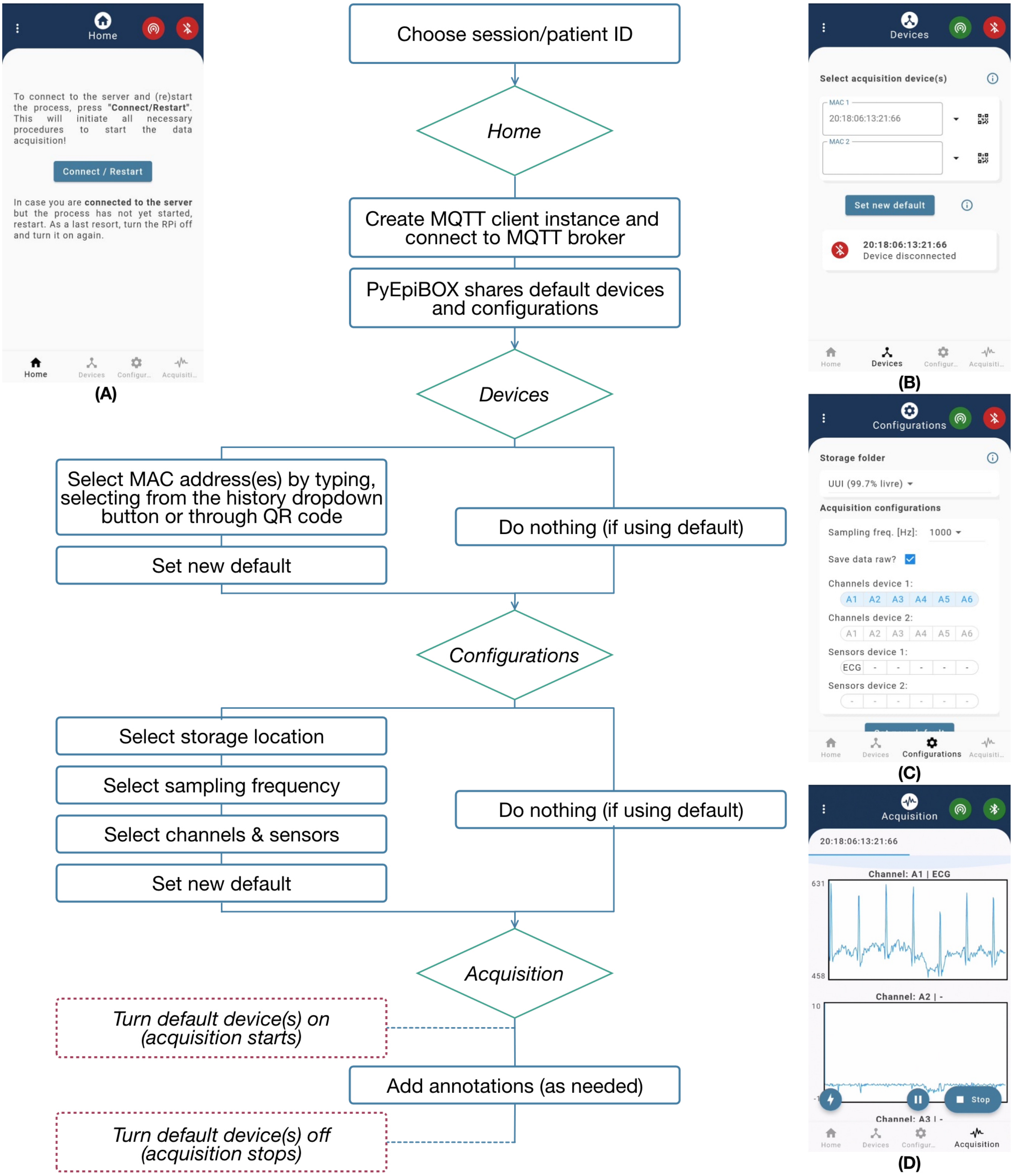

Figure S1. Illustration of a standard interaction between the user and the EpiBOX App.
